# The Mediterranean Diet is Associated with an Improved Quality of Life in Adults with Type 1 Diabetes

**DOI:** 10.3390/nu12010131

**Published:** 2020-01-02

**Authors:** Minerva Granado-Casas, Mariona Martin, Montserrat Martínez-Alonso, Nuria Alcubierre, Marta Hernández, Núria Alonso, Esmeralda Castelblanco, Didac Mauricio

**Affiliations:** 1 Department of Endocrinology and Nutrition, Health Sciences Research Institute & University Hospital Germans Trias i Pujol, 08916 Badalona, Spain; mgranado@igtp.cat (M.G.-C.); marionamarting@gmail.com (M.M.); nalonso32416@yahoo.es (N.A.); 2 Biomedical Research Institute of Lleida (IRBLleida) & University of Lleida, 25198 Lleida, Spain; nurialcubierre@gmail.com; 3 Systems Biology and Statistical Methods for Biomedical Research, Biomedical Research Institute of Lleida (IRBLleida), University of Lleida, 25198 Lleida, Spain; mmartinez@irblleida.cat; 4 Department of Endocrinology and Nutrition, Biomedical Research Institute of Lleida (IRBLleida) & University Hospital Arnau de Vilanova, 25198 Lleida, Spain; martahernandezg@gmail.com; 5 Centre for Biomedical Research on Diabetes and Associated Metabolic Diseases (CIBERDEM), Instituto de Salud Carlos III, 08041 Barcelona, Spain; 6 Department of Endocrinology and Nutrition, University Hospital de la Santa Creu i Sant Pau & Institut d’Investigació Biomèdica Sant Pau (IIB Sant Pau), Autonomous University of Barcelona, 08041 Barcelona, Spain

**Keywords:** Mediterranean Diet, quality of life, treatment satisfaction, type 1 diabetes, dietary pattern, patient-reported outcomes, nutrient intake

## Abstract

This study aimed to assess the potential association between dietary patterns (i.e., the Mediterranean Diet (MedDiet) and healthy eating) and patient-reported quality of life (QoL) and treatment satisfaction (TS) in adults with type 1 diabetes (T1D). A food frequency questionnaire, the Audit of Diabetes-Dependent Quality of Life (ADDQoL-19), and the Diabetes Treatment Satisfaction Questionnaire-status version (DTSQ-s) were administered via personal interviews to 258 participants with T1D. Multivariable analysis showed that a moderate or high adherence to the MedDiet was associated with greater diabetes-specific QoL (β = 0.32, 95% CI = 0.03; 0.61; *p* = 0.029). None of the dietary quality indexes (i.e., the alternate Mediterranean Diet Score (aMED) and the alternate Healthy Eating Index (aHEI)) were associated with the overall TS. However, the aHEI was positively associated with the specific items of TS “convenience” and “flexibility” (β = 0.03, 95% CI = 0.00; 0.06; *p* = 0.042 and β = 0.04; 95% CI = 0.01; 0.06; *p* = 0.011, respectively). On the other hand, the aHEI was negatively associated with the dimension “recommend to others” (β = −0.5, 95% CI = −0.99; −0.02; *p* = 0.042). In conclusion, a moderate and high adherence to the MedDiet was associated with greater QoL. Although neither aMED nor aHEI were associated with the overall TS, some specific items were positively (i.e., “convenience”, “flexibility”) or negatively (“recommend to others”) related to the aHEI. Further research is needed to assess how to improve medical nutrition therapy and its impact on patient-reported outcomes in people with T1D.

## 1. Introduction

People with type 1 diabetes (T1D) need specific treatment, including insulin therapy and advice on physical activity and medical nutrition therapy to ensure optimal self-management of the disease [1]. In Catalonia (Spain), carbohydrate counting and a healthy eating pattern such as the Mediterranean Diet (MedDiet) are included in medical nutrition therapy for people diagnosed with T1D [1,2,3]. This dietary pattern is important for preventing cardiovascular diseases in people with T1D who have a high cardiovascular risk; these people often show an unfavorable lipid profile associated with poorer glycemic control [4].

Fung et al. demonstrated that both the alternate Healthy Eating Index (aHEI) and the alternate Mediterranean Diet score (aMED) were strongly correlated with lower concentrations of biomarkers of inflammation and endothelial dysfunction, related with the development of cardiovascular diseases and diabetes mellitus [5]. Furthermore, both scores focus on dietary patterns rich in fruits and vegetables, whole grains, nuts, fish, and moderate alcohol consumption; therefore, these two indexes are useful measures of dietary patterns and the associated cardiovascular risk. Scientific evidence has shown that the presence of cardiovascular diseases and late diabetic complications have a negative impact on the quality of life (QoL) and treatment satisfaction (TS) of people with T1D [6]. Furthermore, a study performed in children and adolescents (between 13 and 19 years old) described that insulin treatment and dietary limitations in the management of T1D may have several effects on QoL [7].

A recent systematic review of dietary patterns and QoL in older adults with diabetes reported a lack of scientific evidence that relates dietary patterns to QoL in this population [8]. Specifically, the few studies that have looked at the adherence to the MedDiet in relation to QoL and TS found an improved QoL and TS in people with type 2 diabetes who had a higher adherence to the MedDiet [9,10,11]. On the other hand, another study performed in people with diabetes (without identifying the type of diabetes) found a higher health-related quality of life (HRQoL) associated with the combination of multiple healthy lifestyle habits (no smoking, regular physical activity, and a higher intake of fruits and vegetables) [12]. Other cross-sectional studies have only assessed the relationship between TS and adherence to medical nutrition therapy in people with type 2 diabetes [13,14]; they found a higher TS in those who showed a higher adherence to the nutritional recommendations. In adults with T1D, no studies have been designed to assess the potential impact of dietary pattern on QoL and TS. Moreover, no studies have assessed the relationship between the dietary pattern of adults with T1D using dietary quality indexes (i.e., aMED and aHEI) and diabetes-specific QoL instruments. Only one cross-sectional study was performed in adolescents and children (from 13 to 19 years) to assess the relationship between unhealthy lifestyle habits and QoL [7]. However, they found that a combination of unhealthy lifestyle habits (poor adherence to the MedDiet, low physical activity levels, and high sedentary behavior score) was related to a poorer QoL in these participants. Additionally, a case-control study designed to audit diabetes management and QoL in adults with T1D did not find a relationship between dietary pattern and QoL [15]. Other recent studies performed in adults with T1D with celiac disease found a better QoL in those with a higher adherence to a gluten-free diet [16,17]. Finally, a recent meta-analysis of the effectiveness and safety of the possible effect of carbohydrate counting on glycemic control and QoL in people with T1D reported a lack of scientific evidence in terms of QoL and TS [18].

A previously published study revealed that people with type 2 diabetes with a higher adherence to the MedDiet from Catalonia, a Mediterranean region of Spain, had improved QoL and TS [10]. Furthermore, we have previously investigated the dietary pattern and patient-reported outcomes in two studies in people with T1D in this region [19,20]. The first study assessed differences in terms of a MedDiet and healthy eating pattern between participants with and without T1D [19]. The second study assessed the relationship between the presence of diabetic retinopathy and patient-reported outcomes (i.e., QoL and TS) [20]. We found that participants with T1D had a higher adherence to the MedDiet in comparison with a group without diabetes. Additionally, diabetic retinopathy was negatively associated with QoL and TS. Further, we also found that factors related with the lifestyle of the participants (i.e., non-smokers and practice of regular physical activity) were associated with a higher adherence to the MedDiet and a greater QoL. In this context, we hypothesized that as people with T1D receive regular and individual medical nutrition therapy to ensure an optimal management of the disease, improved adherence to the MedDiet and a healthy eating pattern could be associated with better QoL and TS. Thus, the aim of the study was to determine the association between the dietary patterns (i.e., MedDiet and healthy eating) with patient-reported outcomes (i.e., QoL and TS) in people with T1D from a Mediterranean country. To our knowledge, this is the first study specifically designed to assess the relationship between QoL and TS and dietary patterns in adults with T1D.

## 2. Materials and Methods

This was a two-center cross-sectional study. The study participants were individuals diagnosed with T1D who were regularly cared for at the reference hospital and who were from a rural/semi-urban area or an urban area. This is a sub-study of a previously published study performed to assess adherence to the MedDiet between a T1D group and a group without diabetes [19]. From the total sample of 259 participants with T1D included in that study, one participant was excluded because he did not answer all of the questionnaires. Therefore, a final sample of 258 participants with T1D was included. More details of the study design are provided in the previous publication [19]. The inclusion criteria were a diagnosis of T1D with a disease duration of more than one year and a current age older than 18 years. The exclusion criteria were: The presence of psychological or cognitive deterioration; being a healthcare professional; the presence of previous clinical cardiovascular diseases or diabetic foot disease; pregnancy; having advanced complications or conditions that require a specific medical nutrition therapy, such as macroalbuminuria (urine albumin/creatinine ratio > 299 mg/g); and renal insufficiency (estimated glomerular filtration rate < 60 mL/min). The ethics committees of the participating centers (University Hospital Arnau de Vilanova, Lleida, Spain and University Hospital Germans Trias i Pujol, Badalona, Spain) approved the study (PI-13-095 and PI-15-147, respectively), and written informed consent was obtained from all of the study participants.

### 2.1. Clinical Variables

Trained researchers interviewed all of the participants individually, and clinical records were thoroughly reviewed to collect all the relevant study data. Blood and urine samples were collected in the fasting state. Low-density lipoprotein cholesterol was estimated by the Friedewald formula. Glycated hemoglobin (HbA1c) was determined by HPLC (VariantTM, Bio-Rad Laboratories S.A., Madrid, Spain) and its concentrations were expressed in National Glycohemoglobin Standardization Program/Diabetes Control and Complications trial units. Urine albumin was measured with an immunoturbidimetric method and a Roche/Hitachi Modular P analyzer (Roche Diagnostics, Barcelona, Spain). Waist circumference was measured at the midpoint between the lower margin of the least palpable rib and the top of the iliac crest. Hypertension and dyslipidemia were defined by the use of drugs for treating these conditions. Microalbuminuria was defined as an albumin-to-creatinine ratio >30 mg/g. Physical activity was assessed using the method of Bernstein et al., which was designed with people aged 35 to 74 years from Switzerland [21], and validated by Cabrera de León et al. in Spanish people aged 18 to 75 years [22]; participants were classified as performing regular physical activity if they spent at least 4 metabolic equivalents (METs) participating in any activity, such as walking or cycling for more than 25 min/day, and were classified as sedentary if they spent up to 25 min/day. Educational level was classified as lower if the participant did not have a university degree and as a graduate or higher if he or she had a university education. Retinopathy was assessed and classified by an ophthalmologist. Tobacco exposure included current and former smokers.

### 2.2. Dietary Pattern Assessment

A food frequency questionnaire (FFQ) was individually administered by two trained researchers [23,24]. This is a semiquantitative questionnaire that has been validated and adapted for the Spanish population. This was based on the Nurses’ Health Study and was used in our previously published study [19]. The FFQ used was a modified version from a previous FFQ based on the Harvard questionnaire [23]. This version was developed and validated using four 1-week dietary records performed during one year in an adult population in Spain. Individual nutrient intake and food consumption were validated, adjusting for energy intake. Moreover, this FFQ was administered at six years after the baseline assessment and compared with these 1-week dietary records to assess the validity and reproducibility at six years of follow-up. Subsequently, the authors performed a specific validation analysis using nutrients related with pregnancy (i.e., carotenoids, folate, vitamin B12, vitamin C, and α-tocopherol); these were adjusted for energy intake and validated with their concentration in blood specimens [24]. The questionnaire contains 101 items, which are used to record the usual consumption over the previous year prior to the visit [25].

The adherence to the MedDiet was assessed using the aMED score based on the MedDiet scale [26]. The aMED score ranges from 0 (minimal adherence) to 9 (maximal adherence). This includes vegetables, legumes, fruits, nuts, whole grain, red and processed meat, fish, monounsaturated fatty acid-to-saturated fatty acid ratio, and alcohol consumption. Additionally, we determined the aHEI based on the Dietary Guidelines for Americans and the Food Guide Pyramid [5]. This includes vegetables, fruits, nuts, soy, white to red meat ratio, cereal fiber, trans fat, polyunsaturated fatty acid-to-saturated fatty acid ratio, alcohol consumption, and long-term multivitamin use. The score ranges from 0 (lower quality diet) to 80 (higher quality diet), excluding long-term multivitamin use in the Spanish population [19]. Daily nutrient intake was calculated by adjusting for energy intake and was estimated by multiplying the frequency of use for each food by the nutrient composition of the portion/size specified by each individual on the FFQ; these results were added across all foods [24].

### 2.3. Patient-Reported Outcomes

The Audit of Diabetes-Dependent Quality of Life (ADDQoL-19) was administered to assess QoL; this is a disease-specific measure designed and validated for Spanish people with diabetes [27,28,29]. This instrument contains 21 items, of which 19 are related to specific life domains. The impact of diabetes on each domain is weighted according to the importance grade that each participant reported on the QoL. The mean of the 19 specific domains is determined with the average weighted impact (AWI) score. The scores range from +3 (maximum positive impact) to −9 (maximum negative impact). Moreover, five of the 19 items that might not be relevant to some participants are included in a preliminary question that can be ignored if it is not applicable. In this questionnaire, two general items are scored separately; one of them measures the present QoL and ranges from +3 (excellent) to −3 (very bad). The other measures diabetes-specific QoL and ranges from −3 (maximum negative impact) to +1 (maximum positive impact).

TS was measured with the Diabetes Treatment Satisfaction Questionnaire-status version (DTSQ-s), a diabetes-specific questionnaire validated for Spanish people with diabetes, and this questionnaire was individually administered [30,31]. This instrument consists of 8 items scored on a 6-point scale, ranging from 0 (never) to 6 (always). The final score is calculated by adding the scores of the six items and ranges from 36 (very satisfied) to 0 (very unsatisfied). The other two items are calculated separately and measure the perceived frequency of hyperglycemia and hypoglycemia. They are scored from 0 (never) to 6 (always).

### 2.4. Data Analysis

Descriptive analysis included median and interquartile range for quantitative variables and frequency and percentages for qualitative variables. Multivariable linear regression models were fit to explain QoL, as well as TS total scores. Specifically, we modeled six response variables, including present quality of life (QoL), diabetes-specific QoL, and the AWI score (ADDQoL-19 summary score), as well as the perceived frequencies of hyperglycemia/hypoglycemia and TS (DTSQ-s summary score). Initially, simple regression models were fit to assess the statistical significance of the association between each explanatory variable and each outcome. Then, a first multivariable regression model for each outcome was built by starting with all candidate variables with a univariate Wald test *p*-value below 0.20 (showed in the previous simple linear regression models) and testing the deletion of each variable whose exclusion gives the most insignificant impact on the model fit (Wald-test based), and repeating this procedure until all variables in the model showed a statistically significant contribution. Variables out of the multivariable model were assessed and added if they showed a statistically significant contribution (Wald test) on the confounding effect (change in the coefficients of the model higher than 10%). Finally, interactions with diabetes duration and sex were also examined. All statistical analyses were performed with R [32], and a significance level of 0.05 was applied.

## 3. Results

The clinical and sociodemographic characteristics according to aMED and aHEI are shown in Table 1 and Table 2, respectively. The prevalence of retinopathy in the study participants was 42.1%. In addition, they showed a fair lipid profile. In terms of dietary quality index, aHEI was frequently low (75.6%), although a high proportion of participants with low aHEI exhibited a low and moderate adherence to the MedDiet (100.0% and 75.9% for the aMED, respectively; *p <* 0.001). Furthermore, in rural and semi-urban areas, there was a higher proportion of participants with moderate and high adherence to the MedDiet (51.2% and 56.3%, respectively; *p* = 0.010) and healthy eating (71.4% for the aHEI; *p <* 0.001). Participants with T1D and moderate or high aMED scores showed more favorable high-density lipoprotein cholesterol levels (*p* = 0.025) (Table 1). Finally, increasing age was associated with higher aHEI scores (*p* = 0.010) (Table 2). The daily nutrient and food intake of the study group is shown in Tables S1 and S2 of the Supplementary Materials.

### 3.1. Quality of Life and Dietary Pattern

Regarding the dietary quality index, the multivariable analysis showed that moderate and high adherence to the MedDiet were associated with greater diabetes-related QoL (*p* = 0.029) after adjusting for age, tobacco exposure, and physical activity (Table 3). However, neither of the dietary quality indexes (i.e., aMED and aHEI) were related to the current QoL and AWI scores. No association was found between the dietary quality indexes and other items of the ADDQoL-19 questionnaire.

### 3.2. Treatment Satisfaction and the Mediterranean Diet

In the multivariable analysis, none of the dietary quality measures were related to global TS (Table 4). However, aHEI was positively associated with some items of DTSQ-s, i.e., “convenience” (*p* = 0.042) and “flexibility” (*p* = 0.011); the latter depended on the interaction between diabetes duration and aHEI (*β* = −0.01, 95% CI = 0.00; 0.00, *p* = 0.048). On the other hand, a high adherence to a healthy eating pattern was negatively associated with the dimension “recommend to others” (this specific item assesses the recommendation of this treatment to someone with a diabetes condition similar to yours) (*p* = 0.042). No associations were observed with the other TS items.

## 4. Discussion

Our results indicate that moderate and high adherence to the MedDiet was associated with greater diabetes-related QoL in participants with T1D. However, none of the dietary quality indexes were associated with the overall TS. The aHEI was positively associated with “convenience” and “flexibility” items of the TS questionnaire; however, the aHEI was negatively associated with the dimension “recommend to others”.

There is only one cross-sectional study that has been performed with young people with T1D (from 13 to 19 years old) that associated diabetes-specific QoL with the MedDiet pattern [7]. The researchers did not find any relationship between the MedDiet and QoL in this population, a finding that is in contrast with our results. However, this may be due to a smaller sample size of participants with T1D and a lower dietary quality index in that study (*n* = 31). However, a few studies have assessed the relationship between the MedDiet and QoL in people with type 2 diabetes [9,10,11]. Toobert et al. [9] and Galilea-Zabalza et al. [11] found an improved HRQoL with a higher adherence to the MedDiet in people with type 2 diabetes using generic instruments to measure QoL. Furthermore, similar results were observed in a previous study performed by our group in people with type 2 diabetes using a diabetes-specific questionnaire [10]. Finally, in line with the current results, a cross-sectional study showed an improved HRQoL in people with diabetes with a healthy lifestyle (type of diabetes was not specified) [12].

In terms of TS, we could not identify any study that assessed the relationship between dietary pattern and TS in people with T1D. Our group performed a previous study in people with type 2 diabetes and found an association of greater TS with higher adherence to the MedDiet [10]. Two cross-sectional studies performed in people with type 2 diabetes only found a positive relationship between TS and the adherence to medical nutrition therapy [13,14]; however, despite observing a relationship between aHEI and some items of TS in this study, we did not find any association between the dietary pattern and the overall TS. This could be due to the small number of participants included in our study with a poorer TS final score. Furthermore, these differences between the published studies and our results could be explained, in part, by the fact of people with T1D are treated with intensive insulin therapy from the onset of the disease. However, people with type 2 diabetes are treated with diet, hypoglycemic agents, or insulin depending on their glycemic control, other aspects of the management of the disease and the presence of complications. The relationship between healthy dietary patterns and TS could be due to the fact that diet and nutritional recommendations based on the MedDiet are included in the treatment of people with T1D.

This study has some limitations. This is a sub-study of a previously published study specifically designed to assess the degree of adherence to the MedDiet in people with T1D [19]. Due to the cross-sectional study design, causal relationships between the variables cannot be established. Moreover, changes in lifestyle over time cannot be assessed in this study design. However, this study has several strengths. This is the first study designed to assess the relationship between dietary patterns and patient-reported outcomes, such as QoL and TS, in adults with T1D. Furthermore, the large sample size and the multicenter design, with a well-defined sample, allow us to account for the variability in different geographical areas (a rural/semi-urban and an urban population) of the same region. Additionally, the FFQ used has been shown to be representative of the previous five-year period of food and nutrient intake [33]. The final conclusions are potentially interesting in this research field, as this is the first study to assess the relationship between dietary pattern (Mediterranean Diet and healthy eating) and patient-reported outcomes in adults with T1D.

## 5. Conclusions

A moderate and higher adherence to the Mediterranean Diet was associated with greater QoL in participants with T1D. On the other hand, none of the dietary quality measures (i.e., aMED and aHEI) were associated with global TS, although a healthier eating pattern was related with some specific items. Further research is needed in this area to establish new approaches focused on the medical nutrition therapy and their influence on the quality of life and treatment satisfaction of people with T1D. In addition, a causal relationship and definitive conclusions would be necessarily determined by future randomized clinical trials.

## Figures and Tables

**Table 1:** Descriptive analysis of the characteristics of the study participants according to the alternate Mediterranean Diet score.

	All (*n* = 258)	Low (0–2) (*n* = 60)	Moderate (3–5) (*n* = 166)	High (6–9) (*n* = 32)	*p*
Sex (men)	117 (45.3)	30 (50.0)	77 (46.4)	10 (31.3)	0.206
Age (years)	43.0 [36.0; 50.0]	41.5 [35.0; 49.0]	43.0 [37.0; 50.0]	46.0 [37.8; 51.8]	0.240
Ethnicity (Caucasian)	254 (98.4)	58 (96.7)	164 (98.8)	32 (100.0)	0.436
Site					0.010
Rural and semi-urban area	121 (46.9)	18 (30.0)	85 (51.2)	18 (56.2)	
Urban area	137 (53.1)	42 (70.0)	81 (48.8)	14 (43.8)	
Educational level ^1^					0.462
Lower	185 (74.9)	47 (81.0)	115 (72.8)	23 (74.2)	
Graduate or higher	62 (25.1)	11 (19.0)	43 (27.2)	8 (25.8)	
Regular physical activity	186 (72.1)	38 (63.3)	121 (72.9)	27 (84.4)	0.093
Tobacco exposure	128 (49.6)	30 (50.0)	79 (47.6)	19 (59.4)	0.474
BMI (kg/m^2^)	25.0 [22.6; 27.6]	25.0 [22.0; 28.3]	25.2 [22.8; 27.4]	24.7 [22.2; 26.9]	0.686
Waist (cm)	88.0 [79.0; 96.2]	89.0 [81.0; 99.0]	88.0 [79.0; 96.0]	87.0 [75.0; 95.5]	0.397
Retinopathy	102 (42.1)	23 (38.3)	66 (39.8)	13 (40.6)	0.949
Microalbuminuria	19 (7.4)	6 (10.0)	12 (7.2)	1 (3.1)	0.552
Hypertension	64 (24.8)	11 (18.3)	45 (27.1)	8 (25.0)	0.403
Dyslipidemia	102 (39.5)	24 (40.0)	67 (40.4)	11 (34.4)	0.815
Diabetes duration (years)	20.0 [14.0; 29.0]	20.5 [15.0; 27.8]	20.0 [14.0; 29.0]	20.5 [14.8; 30.0]	0.785
HbA1c (%)	7.4 [7.0; 8.0]	7.5 [7.1; 8.1]	7.4 [6.9; 8.0]	7.3 [7.0; 8.1]	0.857
HbA1c (mmol/mol)	57.4 [53.0; 63.9]	57.9 [53.8; 64.5]	57.4 [52.2; 63.9]	56.3 [53.0; 65.3]	0.857
Total cholesterol (mg/dL)	176.0 [162.0; 200.0]	174.0 [160.0; 193.0]	176.0 [162.0; 200.0]	186 [167.0; 202.0]	0.459
LDL-c (mg/dL)	100.0 [85.7; 115.0]	102.0 [85.2; 116.0]	98.1 [84.2; 115.0]	103.0 [90.3; 114.0]	0.711
HDL-c (mg/dL)	63.0 [53.0; 75.0]	57.5 [48.5; 72.2]	63.5 [54.0; 75.8]	69.0 [60.0; 73.8]	0.025
Triglycerides	65.0 [52.0; 83.8]	66.0 [52.8; 83.5]	67.5 [53.0; 85.0]	56.5 [47.8; 69.2]	0.197
aHEI					<0.001
Low (<45)	195 (75.6)	60 (100.0)	126 (75.9)	9 (28.1)	
High (≥45)	63 (24.4)	0 (0.0)	40 (24.1)	23 (71.9)	
aMED	4.0 [3.0; 5.0]	2.0 [1.0; 2.0]	4.0 [3.0; 5.0]	6.0 [6.0; 7.0]	<0.001

Data are shown as the median [interquartile range] or *n* (%). ^1^ There were 11 cases with missing information for educational level. Tobacco exposure, current or former smoker; BMI, body mass index; HbA1c, glycated hemoglobin; LDL-c, low-density lipoprotein cholesterol; HDL-c, high-density lipoprotein cholesterol; aHEI, alternate Healthy Eating Index; aMED, alternate Mediterranean Diet Score.

**Table 2:** Descriptive analysis of the characteristics of the study participants according to the alternate Healthy Eating Index.

	All (*n* = 258)	Low (<45) (*n* = 195)	High (≥45) (*n* = 63)	*p*
Sex (men)	117 (45.3)	96 (49.2)	21 (33.3)	0.040
Age (years)	43.0 [36.0; 50.0]	42.0 [36.0; 49.0]	48.0 [38.5; 54.0]	0.010
Ethnicity (Caucasian)	254 (98.4)	191 (97.9)	63 (100.0)	0.575
Site				<0.001
Rural and semi-urban area	121 (46.9)	76 (39.0)	45 (71.4)	
Urban area	137 (53.1)	119 (61.0)	18 (28.6)	
Educational level ^1^				0.436
Lower	185 (74.9)	135 (73.4)	50 (79.4)	
Graduate or higher	62 (25.1)	49 (26.6)	13 (20.6)	
Regular physical activity	186 (72.1)	135 (69.2)	51 (81.0)	0.101
Tobacco exposure	128 (49.6)	95 (48.7)	33 (52.4)	0.718
BMI (kg/m^2^)	25.0 [22.6; 27.6]	25.2 [22.9; 27.8]	24.6 [22.3; 27.1]	0.277
Waist (cm)	88.0 [79.0; 96.2]			
Retinopathy	102 (42.1)	78 (40.0)	24 (38.1)	1.000
Microalbuminuria	19 (7.4)	15 (7.7)	4 (6.3)	1.000
Hypertension	64 (24.8)	45 (23.1)	19 (30.2)	0.335
Dyslipidemia	102 (39.5)	79 (40.5)	23 (36.5)	0.677
Diabetes duration (years)	20.0 [14.0; 29.0]	20.0 [14.0; 27.5]	23.0 [15.0; 30.0]	0.090
HbA1c (%)	7.4 [7.0; 8.0]	7.4 [7.0; 8.0]	7.5 [7.0; 8.0]	0.911
HbA1c (mmol/mol)	57.4 [53.0; 63.9]	57.4 [53.0; 63.9]	58.5 [53.0; 63.9]	0.911
Total cholesterol (mg/dL)	176.0 [162.0; 200.0]	174.0 [160.0; 200.0]	187.0 [166.0; 202.0]	0.170
LDL-c (mg/dL)	100.0 [85.7; 115.0]	99.0 [84.0; 115.0]	103.0 [89.5; 120.0]	0.175
HDL-c (mg/dL)	63.0 [53.0; 75.0]	62.0 [52.0; 74.0]	64.0 [57.5; 77.5]	0.050
Triglycerides	65.0 [52.0; 83.8]	67.0 [52.5; 85.0]	59.0 [49.5; 80.5]	0.322
aMED				<0.001
Low (0–2)	60 (23.3)	60 (30.8)	0 (0.0)	
Moderate (3–5)	166 (64.3)	126 (64.6)	40 (63.5)	
High (6–9)	32 (12.4)	9 (4.6)	23 (36.5)	
aHEI	40.0 [37.0; 44.0]	38.0 [35.5; 41.0]	48.0 [46.0; 50.5]	<0.001

Data are shown as the median [interquartile range] or *n* (%). ^1^ There were 11 cases with missing information for educational level. Tobacco exposure, current or former smoker; BMI, body mass index; HbA1c, glycated hemoglobin; LDL-c, low-density lipoprotein cholesterol; HDL-c, high-density lipoprotein cholesterol; aHEI, alternate Healthy Eating Index; aMED, alternate Mediterranean Diet Score.

**Table 3:** Multivariable analysis of the Audit Diabetes-Dependent Quality of Life (ADDQoL-19) questionnaire with the alternate Mediterranean Diet Score and alternate Healthy Eating Index.

Items	aMED	β (95% CI)	*p*	aHEI	β (95% CI)	*p*
Present QoL ^a^	>2	0.05 (−0.23; 0.33)	0.742	>44	−0.23 (−0.52; 0.05)	0.103
Diabetes-specific QoL ^b^	>2	0.32 (0.03; 0.61)	0.029	-	0.02 (0.00; 0.04)	0.055
AWI ^c^	>2	0.08 (−0.31; 0.48)	0.680	>44	0.00 (−0.39; 0.39)	0.992

^a^ Adjusted by age and retinopathy. ^b,c^ Adjusted by age, tobacco exposure, and physical activity. aMED, alternate Mediterranean Diet score; AWI, average weighted impact score; aHEI, alternate Healthy Eating Index; CI, confidence interval; QoL, quality of life.

**Table 4:** Multivariable analysis of the Diabetes Treatment Satisfaction Questionnaire-status (DTSQ-s) with the alternate Mediterranean Diet Score and alternate Healthy Eating Index.

Items	aMED	β (95% CI)	*p*	aHEI	β (95% CI)	*p*
Hyperglycemia frequency perception ^a^	>2	−0.33 (−0.74; 0.08)	0.118	>44	−0.07 (−0.47; 0.34)	0.743
Hypoglycemia frequency perception ^b^	>2	−0.41 (−0.83; 0.02)	0.060	>44	−0.26 (−0.69; 0.17)	0.231
Convenience ^c^	-	0.08 (−0.04; 0.21)	0.194	-	0.03 (0.00; 0.06)	0.042
Flexibility ^d^	-	0.06 (−0.06; 0.17)	0.328	-	0.04 (0.01; 0.06)	0.011
Recommend to others ^e^	>2	−0.11 (−0.61; 0.39)	0.668	>44	−0.5 (−0.99;−0.02)	0.042
Final score ^f^	>2	0.39 (−1.21; 2.00)	0.629	>44	0.31 (−1.29; 1.90)	0.706

**^a^** Adjusted by glycated hemoglobin. ^b^ Adjusted by sex and retinopathy. ^c^ Adjusted by sex and tobacco exposure. ^d^ Adjusted by diabetes duration and the interaction between diabetes duration and aHEI or aMED. ^e^ Adjusted by hypertension and diabetes duration. ^f^ Adjusted by diabetes duration. aHEI, alternate Healthy Eating Index; aMED, alternate Mediterranean Diet Score; CI, confidence interval.

## Supplementary Materials

The following are available online at https://www.mdpi.com/2072-6643/12/1/131/s1: Table S1. Daily food intake of the study group; Table S2. Daily nutritional intake of the study group.
