# Supplementary material for: The Mediterranean Diet is Associated with an Improved Quality of Life in Adults with Type 1 Diabetes
Source: Nutrients. 2020 Jan 2;12(1):131. doi: 10.3390/nu12010131 (PMC7020074; doi:10.3390/nu12010131)
Supplement: Supplementary file 1 [file nutrients-12-00131-s001.pdf]

Supplementary Materials

Table S1. Daily food intake of the study group.

Daily food intake (g/day) [a]	T1D (*n* = 258)
Dairy products	368.0 [226.0; 548.0]
Eggs	20.8 [17.5; 23.7]
White meat	34.8 [18.2; 48.7]
Red meat	43.4 [24.9; 67.8]
Processed meat	37.3 [24.4; 56.3]
Meat	124.0 [93.4; 157.0]
Lean fish	21.2 [14.0; 43.6]
Fatty fish	29.5 [15.9; 48.7]
Seafood	7.5 [4.5; 12.7]
Fish	68.1 [45.6; 100.0]
Fruits	209.0 [136.0; 308.0]
Vegetables	228.0 [162.0; 305.0]
Nuts	5.0 [0.9; 13.4]
Legumes	23.2 [15.6; 53.2]
Cereals and pasta	75.9 [48.4; 111.0]
Potatoes	50.6 [28.4; 70.8]
Bread	99.1 [62.7; 135.0]
Sweets	9.8 [2.6; 24.2]
Vegetable fats	39.1 [27.9; 51.4]
Animal fats	0.0 [0.0; 0.0]
Alcohol drinks	26.8 [0.0; 102.0]
Non-alcoholic beverages	1468.0 [1168.0; 1848.0]
Coffee and tea	461.0 [200.0; 540.0]
Prepared meals	44.3 [19.0; 96.5]
Salt	0.2 [0.0; 1.6]

[a] Adjusted by energy intake. Data are shown as median [interquartile].

Table S2. Daily nutritional intake of the study group.

Daily nutrient intake (unit/day) [a]	T1D (*n* = 258)
Energy intake (Kcal)	2075.1 ± 514.1
Carbohydrate (g)	191.0 [175.0; 210.0]
Complex carbohydrate (g)	87.9 [76.7; 100.0]
Sugar (g)	78.6 [64.8; 95.4]
Fiber (g)	22.2 [18.8; 26.2]
Soluble fiber (g)	3.4 [2.7; 4.3]
Insoluble fiber (g)	12.9 [10.4; 15.8]
Glycemic index (%)	82.5 [73.9; 93.1]
Protein (g)	97.6 ± 14.8
Total fat (g)	103.0 ± 15.3
Saturated fatty acids (g)	26.1 [23.2; 29.1]
Mono-unsaturated fatty acids (g)	52.2 ± 10.6
Poly-unsaturated fatty acids (g)	16.2 [14.6; 18.4]
Omega 3 (g)	1.6 [1.4; 1.8]
Omega 6 (g)	14.4 [12.7; 16.6]
Trans fat (g)	1.0 [0.7; 1.2]
Cholesterol (mg)	286.0 [250.0; 321.0]
Palmitic acid (g)	16.2 [14.5; 17.7]
Stearic acid (g)	6.0 ± 1.1
Oleic acid (g)	49.6 ± 10.4
Linoleic acid (g)	14.4 [12.6; 16.5]

Linolenic acid (g)	1.2 [1.0; 1.2]
Arachidonic acid (g)	0.2 [0.1; 0.2]
Eicosapentaenoic acid (g)	0.1 [0.1; 0.2]
Docosahexaenoic acid (g)	0.3 [0.2; 0.4]
Alcohol (g)	2.3 [0.0; 6.9]
Caffeine (g)	188.0 [62.3; 420.0]
Water (g)	2907.0 ± 689.0
Vitamin A (μg)	1105.0 [802.0; 1492.0]
Retinol (μg)	241.0 [183.0; 336.0]
Carotene (μg)	761.0 [534.0; 1115.0]
α carotene (μg)	505.0 [234.0; 853.0]
β carotene (μg)	4142.0 [2952.0; 6088.0]
B cryptoxanthin (μg)	272.0 [166.0; 431.0]
Lutein+zeoxanthin (μg)	3065.0 [2286.0; 6150.0]
Lycopene (μg)	4311.0 [2982.0; 5579.0]
Folate (μg)	277.0 [237.0; 329.0]
Vitamin B_{12} (mg)	7.8 [6.2; 10.0]
Vitamin B_6 (mg)	1.9 [1.6; 2.2]
Vitamin C (mg)	103.0 [72.1; 143.0]
Vitamin D (mg)	4.0 [3.2; 5.1]
Vitamin E (mg)	14.2 [12.7; 16.4]
Thiamine (mg)	1.6 [1.4; 1.7]
Riboflavin (mg)	2.2 [1.9; 2.6]
Niacin (mg)	27.1 [23.7; 31.4]
Niacin equivalents (mg)	43.3 [38.2; 48.1]
Calcium (mg)	1093.0 [895.0; 1324.0]
Iron (mg)	12.7 [11.4; 14.1]
Sodium (mg)	3403.0 ± 559.0
Potassium (mg)	3406.0 ± 670.0
Magnesium (mg)	407.0 [358.0; 466.0]
Zinc (mg)	11.6 ± 1.6
Selenium (μg)	142.0 [125.0; 158.0]

[a] Adjusted by energy intake. Data are shown as mean ± standard deviation (SD) or median [interquartile].
